# Unearthing Shifts in Microbial Communities Across a Soil Disturbance Gradient

**DOI:** 10.3389/fmicb.2022.781051

**Published:** 2022-05-24

**Authors:** Taylor J. Seitz, Ursel M. E. Schütte, Devin M. Drown

**Affiliations:** ^1^Department of Biology and Wildlife, University of Alaska Fairbanks, Fairbanks, AK, United States; ^2^Institute of Arctic Biology, University of Alaska Fairbanks, Fairbanks, AK, United States

**Keywords:** soil disturbance, microbiome, boreal forest, community function, climate change

## Abstract

Permafrost, an important source of soil disturbance, is particularly vulnerable to climate change in Alaska where 85% of the land is underlained with discontinuous permafrost. Boreal forests, home to plants integral to subsistence diets of many Alaska Native communities, are not immune to the effects of climate change. Soil disturbance events, such as permafrost thaw, wildfires, and land use change can influence abiotic conditions, which can then affect active layer soil microbial communities. In a previous study, we found negative effects on boreal plants inoculated with microbes impacted by soil disturbance compared to plants inoculated with microbes from undisturbed soils. Here, we identify key shifts in microbial communities altered by soil disturbance using 16S rRNA gene sequencing and make connections between microbial community changes and previously observed plant growth. Additionally, we identify further community shifts in potential functional mechanisms using long read metagenomics. Across a soil disturbance gradient, microbial communities differ significantly based on the level of soil disturbance. Consistent with the earlier study, the family *Acidobacteriaceae*, which consists of known plant growth promoters, was abundant in undisturbed soil, but practically absent in most disturbed soil. In contrast, *Comamonadaceae*, a family with known agricultural pathogens, was overrepresented in most disturbed soil communities compared to undisturbed. Within our metagenomic data, we found that soil disturbance level is associated with differences in microbial community function, including mechanisms potentially involved in plant pathogenicity. These results indicate that a decrease in plant growth can be linked to changes in the microbial community and functional composition driven by soil disturbance and climate change. Together, these results build a genomic understanding of how shifting soil microbiomes may affect plant productivity and ecosystem health as the Arctic warms.

## Introduction

Across the sub-arctic and arctic regions, the warming climate is rapidly affecting Alaska’s ecosystems through shifts in disturbance regimes, including increased fire and permafrost thaw (Chapin et al., 2008; Johnstone et al., 2010; Schuur et al., 2015; Chasmer and Hopkinson, 2017). Boreal forests, which represent 90% of the world’s forests, are a complex mosaic of coniferous trees and peatlands (Wolken et al., 2011). Much of the boreal forests across Alaska are underlain by discontinuous permafrost that is not immune to the pressures of climate change. Permafrost thaw is associated with direct and indirect changes in plant communities due to significant shifts in soil hydrology which in turn affect nutrient availability and carbon dynamics (Schuur et al., 2007; Yang et al., 2013; Inglese et al., 2017; Sewell et al., 2020), yet only few studies have explored the biotic mechanism contributing to changes in plant community with thaw (Hewitt et al., 2016; Schütte et al., 2019; Seitz et al., 2021).

Permafrost thaw leads to rapid changes in microbial community composition and function, including shifts in several metabolites and genes involving nitrogen and carbon cycling in response to permafrost thaw, and during a thaw event, community function within permafrost quickly converges to that of the active layer (Mackelprang et al., 2011; Coolen and Orsi, 2015; Monteux et al., 2018; Johnston et al., 2019; Messan et al., 2020; Saidi-Mehrabad et al., 2020). Consistent with the pattern of permafrost thaw affecting soil microbial communities, Inglese et al. (2017) found that active layer detachments, a form of permafrost disturbance, significantly affect fungal and Archaeal community composition of the active layer. They observed greater proportions of Nitrososphaerales, an ammonia oxidizing Archaea, in disturbed communities compared to undisturbed, again indicating a shift in nitrogen cycling following permafrost thaw. Within fungal communities, Inglese et al. (2017) saw a large decrease in ericoid mycorrhizal fungi and Ascomycota overall in disturbed soils compared to undisturbed communities. These groups are often found associated with arctic ericoid plant species. These authors suggest that both the increase of ammonia-oxidizing Archaea and the reduction in hyphal fungi could exacerbate further permafrost thaw and landscape change in the arctic.

In a previous study, we found that increased soil disturbance was associated with decreased plant productivity across multiple key boreal plant species, and that this decrease may be mediated by underlying differences in soil microbial community composition (Seitz et al., 2021). The microbial community analysis in Seitz et al. (2021) was based on long read sequence analysis using Oxford Nanopore sequencing technology. However, the usefulness of long read sequence data is not yet well established for environmental studies. Here, we had the unique opportunity to determine whether 16S rRNA gene sequencing would show consistent differences in community composition compared to the previously obtained nanopore sequences, as well as increase our understanding of how disturbance affects alpha and beta diversity metrics. In this study, we combined microbial community composition and functional analysis, and linked belowground changes with changes in plant productivity, which statistically results in a comprehensive understanding of disturbance-induced changes in belowground processes affecting plant communities.

In this study, we determined differences in microbial community composition and function across the disturbance gradient using both 16S rRNA gene sequencing and long read metagenomic sequencing based on nanopore sequencing technology. Further, we used linear regression analysis to determine whether within disturbance level variation can predict plant productivity previously observed in the plant growth experiment (Seitz et al., 2021). We hypothesized that both alpha and beta diversity would increase with disturbance, and furthermore, not only would microbial community composition differ across disturbance levels, but also potential community function. The data of this study significantly add to our previous study (Seitz et al., 2021) providing a more comprehensive understanding of how disturbances affect belowground processes and that these changes in microbial community composition and function indeed significantly impact plant communities.

## Materials and Methods

### Sample Site Description

The soil samples we collected and used for this study were previously described in Seitz et al., 2021. We collected samples from interior Alaska from the Fairbanks Permafrost Experiment Station (FPES; 64.877°N, 147.670°W). This forested site was established and disturbed in 1946 as part of the United States Army Corps of Engineers Cold Regions Research and Engineering Laboratory, which aimed to simulate and study how disturbance events influence the ecology of the boreal forest. FPES contains three, 3,721 m^2^ Linell plots, described in Linell, 1973 of varying degrees of soil disturbance. When FPES was established, the first plot, referenced as undisturbed (UD), was left untouched. The second plot (semi-disturbed, SD) was cleared of all above ground vegetation and trees, while roots and organic soil layers stayed undamaged. The third plot (most disturbed, MD) was stripped entirely of surface level vegetation and organic material until the mineral soil was reached. Since the original disturbance event, permafrost in the SD plot degraded to 4.7 m below the surface soils, and the permafrost in the MD plot degraded down to 9.8 m below the surface (Douglas et al., 2008).

Fairbanks Permafrost Experiment Station is an example of a Taiga boreal forest found in subarctic regions across the world. The untouched UD plot is characterized by a thick covering of black spruce (*Picea Mariana*) with white spruce (*Picea glauca*) scattered throughout. The UD understory is largely dominated by low-bush cranberry (lingonberry, *Vaccinium vitis-idaea*) and Labrador tea (*Rhododendron groendlandicum*), and the ground is continuously covered with *Sphagnum* and feather moss, and lichen. The SD plot is characterized by a highly mixed stand of black spruce, white spruce, Alaskan birch (*Betula neoalaskana*), and willow (*Salix* spp.). SD trees are taller than those in the MD plot which is characterized by young willow, black spruce, and Alaskan birch (Douglas et al., 2008).

### Sample Collection and DNA Extraction

To assess how soil microbial communities are shifting based on previous soil disturbance, we collected a total of 48 soil samples on 28 May 2018, with 16 each from UD, SD, and MD plots. The collection of these cores and their original use was described in Seitz et al. (2021). In brief, we extracted cores from a selection of quadrats throughout each disturbance plot to sample the within disturbance level heterogeneity. Using sterile technique, we removed the top layer of moss at each sampling point and then collected the top layer of soil using a 4.5 cm diameter by 10 cm height soil corer. We stored samples in a cooler throughout the duration of sample collection before they were transported back to the lab. After the samples arrived at the lab, we mechanically homogenized each individual soil core and stored them at 4°C.

After homogenizing each soil sample, we extracted DNA from 0.25 g of soil using the Qiagen PowerSoil Kit (Qiagen Inc., United States) according to the manufacturer’s instructions. We then checked the quality and concentration of DNA using a NanoDrop One spectrophotometer (ThermoFisher Science, United States) and a Qubit 4.0 fluorometer (Fisher Science, United States).

### PCR and 16S rRNA Gene Sequencing and Analyses

We determined microbial community composition based on 16S rRNA gene sequencing, which allowed us to determine taxonomy at a finer resolution compared to our previous study and gave us the unique opportunity to compare the commonly used standard of 16S rRNA gene sequences to the not as well-established long read based on nanopore sequencing (Seitz et al., 2021). After extracting DNA, we performed a 1:10 dilution of all samples to be used for PCR. We amplified the V4 region using dual-indexed 515F (Parada et al., 2016) and 806R (Apprill et al., 2015) primers following the EMP PCR protocol^1^ to be used for 16S rRNA gene amplicon analysis. Following the first round of amplification, we used gel electrophoresis to determine the presence of PCR products. We ran duplicate PCR reactions and then pooled the two reactions for each unique sample. The amplicons were then sequenced on an Illumina MiSeq using v3 reagents at the Institute of Arctic Biology Genomics Core Lab.

Upon obtaining the 16S rRNA amplicon data, we first demultiplexed all reads using Mr_Demuxy (version 1.2.0, https://pypi.org/project/Mr_Demuxy/1.2.0/). We then processed and analyzed the paired-end, demultiplexed reads using the Quantitative Insights into Microbial Ecology (QIIME2) framework (version 2020.8.0). We used the QIIME 2 plugin DADA2^2^ to obtain a table of representative sequences and frequencies, remove low quality regions of reads, and to merge our paired reads for further processing. Next, we constructed a rooted phylogenetic tree from representative sequences using the QIIME 2 alignment plug in and then calculated microbial diversity metrics based on amplicon sequence variants (ASVs) allowing taxonomic classification at a finer resolution compared to the long read data based on nanopore sequencing used in our previous study (Seitz et al.). After constructing the tree, we assigned taxonomy with the QIIME 2 taxonomy classifier plugin (Quast et al., 2012; Bokulich et al., 2018) using a Naïve Bayes classifier pre-trained on the Silva 138 99% OTUS (515F/806R region). We next filtered out any chimeric, mitochondrial, and chloroplast reads from our samples using the QIIME 2 plugin feature table.

### Amplicon Statistical Analyses

We determined alpha and beta diversity, tested whether diversity differed significantly across disturbance levels, and determined whether microbial community composition was significantly associated with changes in plant productivity. This type of analysis was absent in our previous study (Seitz et al., 2021), and 16S rRNA gene sequences enabled us to complete these analyses based on finer taxonomic resolution (ASV). All statistical analyses performed in R utilized version 3.6.1. After obtaining our filtered feature table, we used *qiime2R* (version 0.99.35; Bisanz, 2018) to import our QIIME 2 data and artifacts into R Studio. We converted our taxonomic data into a *phyloseq* (version 1.30.0; McMurdie and Holmes, 2013) object and filtered out one sample with low sampling depth (less than 14,377 reads). We transformed our data into relative abundance measures. We used *vegan* (version 2.5.7; Oksanen et al., 2020) to calculate alpha diversity metrics (Pielou’s evenness and Faith’s phylogenetic diversity) based on ASVs. We visualized our results with *ggplot2* (version 3.3.3; Wickham, 2016).

To test the significance of overall differences between UD, SD, and MD soil communities, we calculated the Bray–Curtis similarity index to compare community beta diversity using amplicon sequence variants (ASVs). We tested the effects of disturbance on community composition using the Permutational Multivariate ANOVA (PERMANOVA) using the “adonis” function in *vegan* (10,000 permutations). We visualized beta diversity differences using nonmetric multidimensional scaling (NMDS) and *ggplot2*.

To visualize community membership across soil samples and taxonomic levels, we generated heatmaps using the R package *gplots* (version 3.1.1; Warnes et al., 2020). At the phylum level, we filtered out any phyla that were not present in samples at higher than 1% relative abundance. When visualizing at the family level, we filtered out any family that was present at a maximum abundance of less than 5% across all samples.

To examine the statistical relationship between microbial community composition and resulting plant productivity Seitz et al. (2021) observed in their growth experiment, we utilized linear regression with the *lm* function in R (R Core Team, 2019). Since the first NMDS axis from our 16S rRNA amplicon analysis describes microbial community variation across soil disturbance level, we extracted the scores from the axis of variation, NMDS1, to test whether community variation within disturbance level explains leaf count and plant height from Seitz et al. (2021). For example, for bog blueberry plants, we performed linear regression using MD community variation NMDS scores and plant height as the growth measure. We repeated the regression using SD and then UD scores. We conducted this series of analyses for each plant type and growth measure (height and leaf count). We then adjusted *p* values using the Benjamini-Hochberg method with a false discovery rate of 0.25 on the basis that follow up studies are relatively low cost (Benjamini and Hochberg, 1995). We then used *ggplot2* (version 3.3.3) to visualize significant relationships between plant productivity and microbial community variation within each soil disturbance level.

Next, we compared community composition estimates between the gold standard 16S rRNA amplicon sequencing and long read nanopore sequencing using the base R package, *stats* (version 3.6.1; R Core Team, 2019). We calculated the Pearson’s correlation coefficient estimates between 16S and long read relative abundances for 10 specific taxonomic groups previously highlighted as biomarkers of either UD or MD communities in Seitz et al. (2021).

### Functional Analysis

We performed community functional analyses on previously collected metagenomic data from our soil cores described in Seitz et al., 2021 (ENA Project: PRJEB42020). Determining microbial function based on the metagenomic data was an aspect lacking in our previous study (Seitz et al., 2021), but essential to obtain a more complete understanding on the effects of disturbances such as permafrost thaw on microbial communities. We utilized the MEGAN-LR (long read) pipeline (Huson et al., 2018) following the parameters described by Arumugam et al. (2019). MEGAN-LR is a tool that performs both taxonomic and functional community classification. Briefly, this pipeline aligns long reads against an NCBI-nr database using DIAMOND (Buchfink et al., 2015) by performing a frame-shift aware DNA-to-protein alignment. The alignments are then processed by MEGAN using an LCA-based algorithm for taxonomic binning and an interval-tree based algorithm for functional binning.

Following classification of potential functions *via* MEGAN-LR, we utilized the MEGAN comparison function to normalize read counts and compare functions across samples. We then extracted the evolutionary genealogy of genes: Non-supervised Orthologous Groups (EGGNOG)-Clusters of Orthologous Group (COG) functions into a matrix. We calculated differences in functional diversity metrics using the *vegan* R package (version 2.5.7; Oksanen et al., 2020) and visualized differences in function based on the identified COG functions using nonmetric dimensional scaling (NMDS) using *ggplot* (version 3.3.3; Wickham, 2016). We tested the effects of disturbance level through a PERMANOVA using the “adonis” function of *vegan*. To identify potential functional indicators of each disturbance level (UD, SD, and MD), we used the R package *indicspecies* (version 1.7.9; De Caceres and Legendre, 2009). In this application, indicator species analysis is using indices of a gene’s or taxon’s (in this case gene) relative abundance and their occurrence to estimate the strength of its association with specific groups (in this instance soil disturbance level). For the indicator analysis, we used only the identified COGs that were identified *via* MEGAN-LR. Within *indicspecies*, we used the function “multipatt” to determine a list of COGs that are significantly associated with each disturbance level. We specified 999 random permutations. We visualized the results using the R package *gplots* (version 3.1.1; Warnes et al., 2020).

## Results

### Microbial Community Composition Differs Based on Soil Disturbance Level

**Figure 1:**
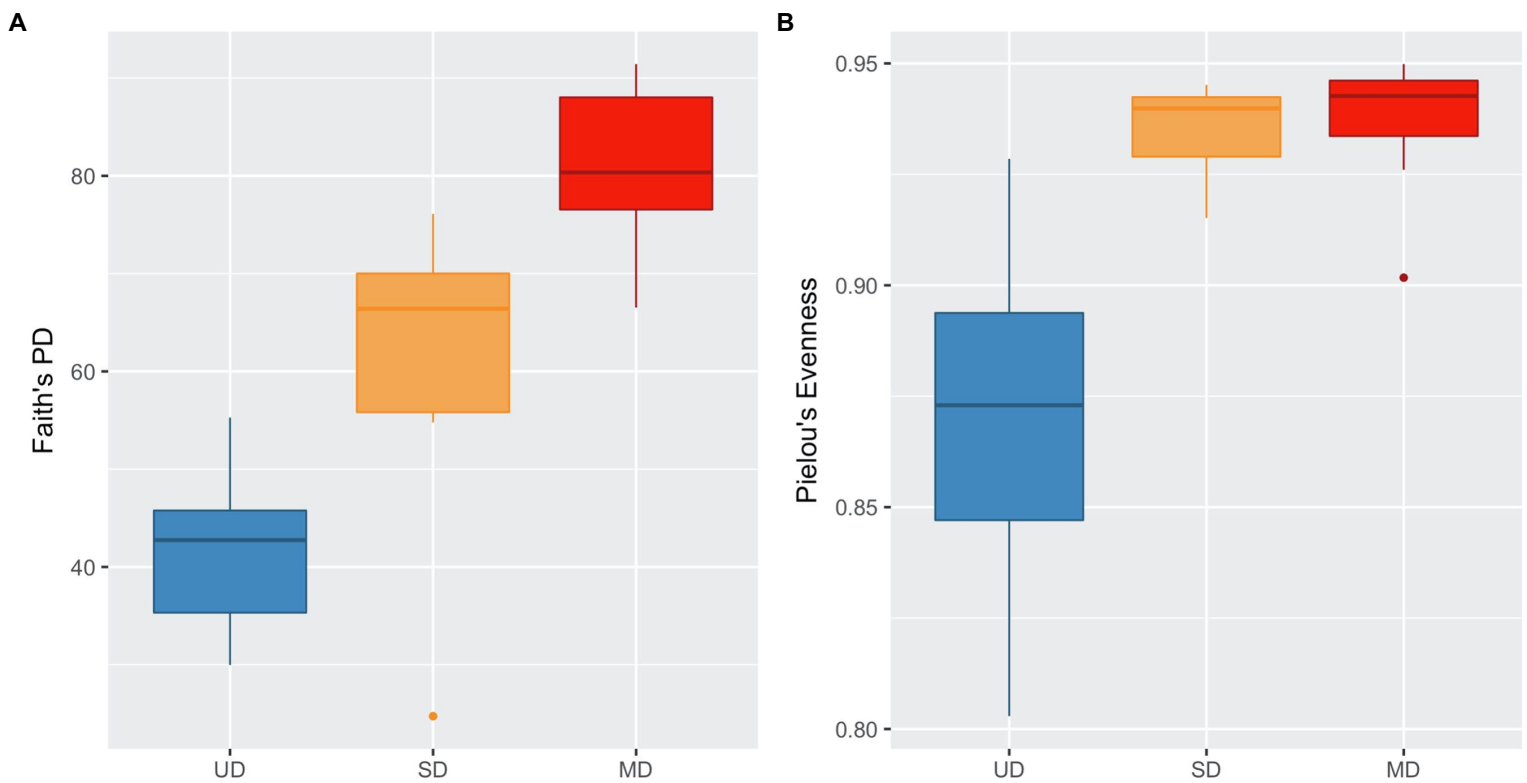
Boxplots of **(A)** Faith’s Phylogenetic Diversity and **(B)** Pielou’s Evenness.

We identified a total of 17,005 ASVs within the 47 retained samples with a feature count ranging from 14,076 to 66,994, and a mean feature count of 50,943. After filtering out ASVs that were not seen more than three times in at least 20% of samples, we detected 1,005 ASVs. Using the filtered ASV data, we compared both alpha and beta diversity to test for differences in bacterial community across soil disturbance level. We found alpha diversity increased significantly within communities as the level of soil disturbance increased (Figure 1). A Kruskal-Wallis test revealed that the Faith’s Phylogenetic Diversity within MD soil communities was significantly higher than in either SD or UD communities (Table 1). We also found that Pielou’s Evenness did not differ significantly between MD and SD communities but did increase from UD to MD and SD soils.

**Table 1:** All groups and pairwise comparison of alpha diversity by Kruskal-Wallis.

	Group 1	Group 2	H value	*p* Value	*q* Value
Evenness	MD	SD	2.626562	0.105089	0.105089
		**UD**	21.840909	0.000003	0.000007
	**SD**	**UD**	21.025	0.000005	0.000007
	**All groups**		30.194481	2.78E−07	
Faith’s PD	**MD**	**SD**	17.226562	0.000033	0.00005
		**UD**	23.272727	0.000001	0.000004
	**SD**	**UD**	16.25625	0.000055	0.000055
	**All groups**		35.637824	1.83E-08	

Bold denotes significance of *p* < 0.05.

**Figure 2:**
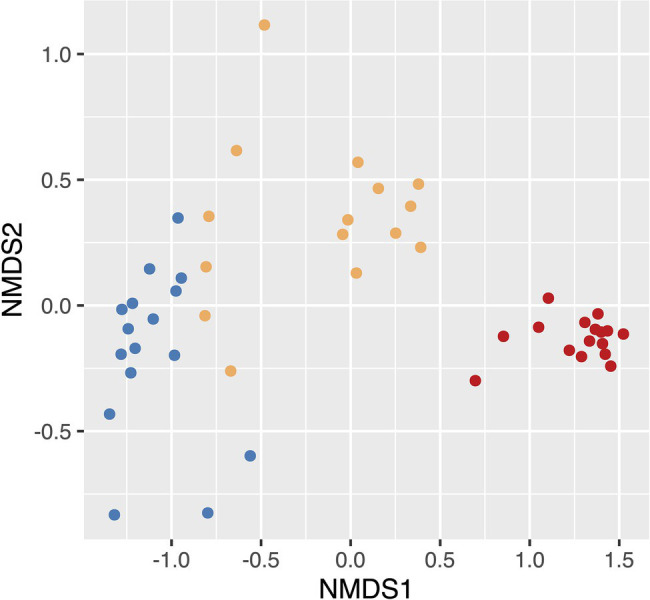
Nonmetric multidimensional scaling (NMDS) based on Bray–Curtis dissimilarity distances showing the differences in beta diversity between undisturbed (UD), semi disturbed (SD), and most disturbed (MD) soil communities. Points colored by the level of Fairbanks Permafrost Experiment Station (FPES) soil disturbance with blue = UD (*n* = 16), gold = SD (*n* = 15), and red = MD (*n* = 16).

We found that microbial community composition differed significantly at the ASV level across the three soil disturbance levels (Figure 2; stress = 0.055; PERMANOVA *F*_2,44_ = 28.682, *p* < 0.001). Communities clustered based on disturbance level along the NMDS first axis. MD communities clustered distinctly from both SD and UD communities, while SD and UD communities displayed some overlap. The variation in microbial community composition along axis 2 was not associated with separation of the microbial communities across the disturbance level groups. To compare the taxonomic results of 16S and metagenomic sequencing, we completed correlation analyses (Pearson’s correlation coefficient) on the relative abundances of biomarkers highlighted in Seitz et al. (2021). We found highly significant positive correlations between 16S and metagenomic relative abundances of eight biomarkers ranging from 0.641 to 0.942 with an average of 0.808 (Table 2). Two biomarkers, the phylum Proteobacteria and the order Myxococcales, did not show significant correlations between sequencing methods.

**Table 2:** Pearson’s correlation estimates (*r*) between 16S and long read relative abundances.

Taxonomic rank	Taxon	Estimate	P value
Phylum	Acidobacteria	0.641225	1.20 × 10^−06^
Phylum	Proteobacteria	0.3013	0.154
Order	Myxococcales	−0.0129658	0.931
Order	Propioniales	0.7038802	3.40 × 10^−08^
Order	Burkholderiales	0.771654	2.17 × 10^−10^
Order	Acidobacteriales	0.8856192	2.20 × 10^−16^
Family	*Acidobacteriaceae*	0.9225034	2.20 × 10^−16^
Family	*Comamonadaceae*	0.7906535	3.82 × 10^−11^
Family	*Micromonasporaceae*	0.9424725	2.20 × 10^−16^

**Figure 3:**
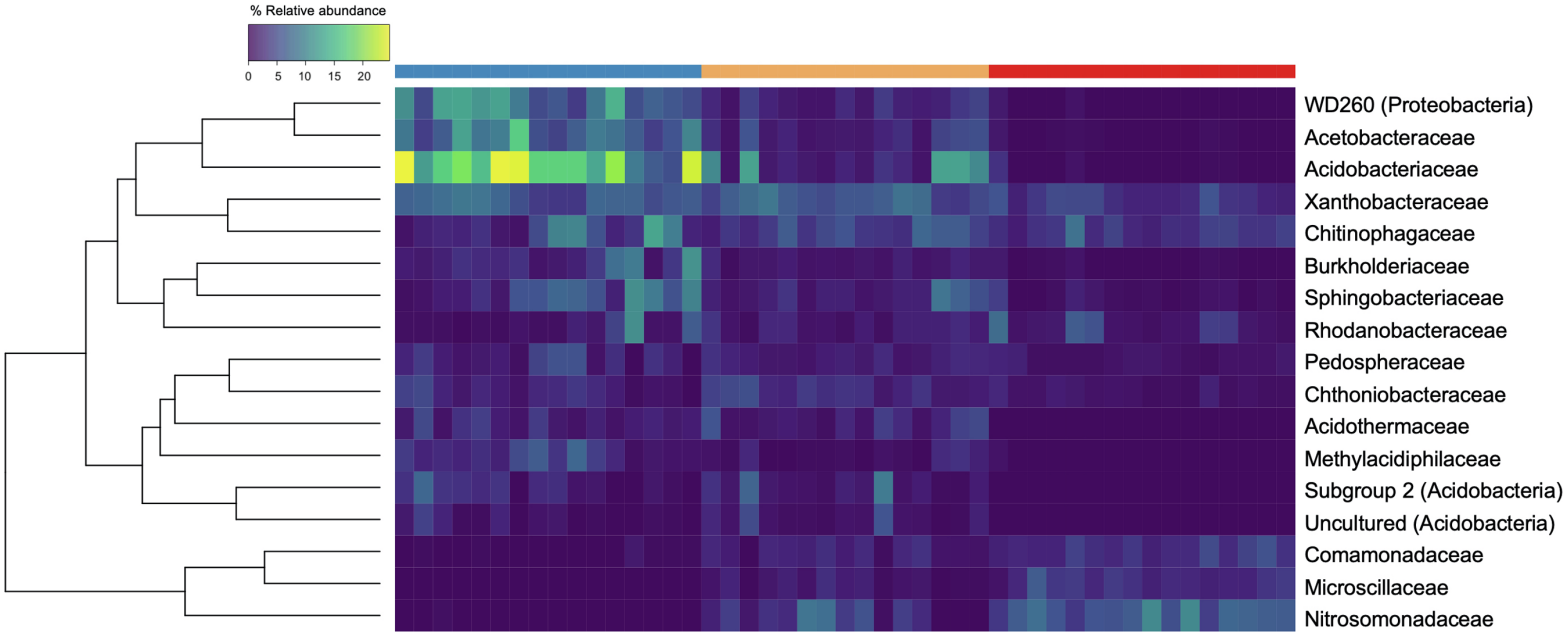
Heatmap of bacterial families present at maximum abundances greater than 5%. Each row corresponds to a family, and each column corresponds to an individual soil core community. The top row signifies the level of soil disturbance of each core with blue = undisturbed (UD; *n* = 16), gold = semi disturbed (SD; *n* = 15), and red = most disturbed (MD; *n* = 16). The rows are clustered by Bray-Curtis distance and the color of each box displays the relative abundance of the taxon within that core.

Across all three disturbance levels, we observed shifts in community membership and abundances. We identified 11 phyla that were present at maximum abundances greater than 5% within all samples. Out of those 11 phyla, Proteobacteria were the most abundant phylum, present at mean relative abundances of 40.7% (range 25.2%–58.8%) in UD, 36.1% (range 27.5%–43.8%) in SD, and 38.0% (range 29.9%–46.4%) in MD soil communities. Looking further at the taxonomic composition of the soil communities, we found 17 groups classified to the family level that were present at maximum abundances greater than 5% (Figure 3). Of those, *Acidobacteriaceae* was the most abundant family in UD, present at a mean relative abundance of 16.5% (range 5.46%–24.7%) compared to a mean of 0.33% (range 0.00%–3.21%) in MD communities. The most abundant families found in MD communities were *Comamonadaceae* and *Nitrosomonadaceae*, both Proteobacteria, present at mean relative abundances of 3.33% (range 2.0%–5.7%) and 6.53% (range 3.8%–10.3%), respectively. Within UD communities, *Comamonadaceae* and *Nitrosomonadaceae* were present at mean relative abundances of 0.28% (range 0.03%–1.57%) and 0.05% (range 0.00%–0.51%), respectively. Within the phylum Acidobacteria, Subgroup 2 and “uncultured” bacteria, and WD260, a group of uncultured bacteria belonging to the phylum Proteobacteria were present at relative abundances greater than 5% throughout our samples.

### Microbial Community Composition as a Predictor for Plant Growth

**Figure 4:**
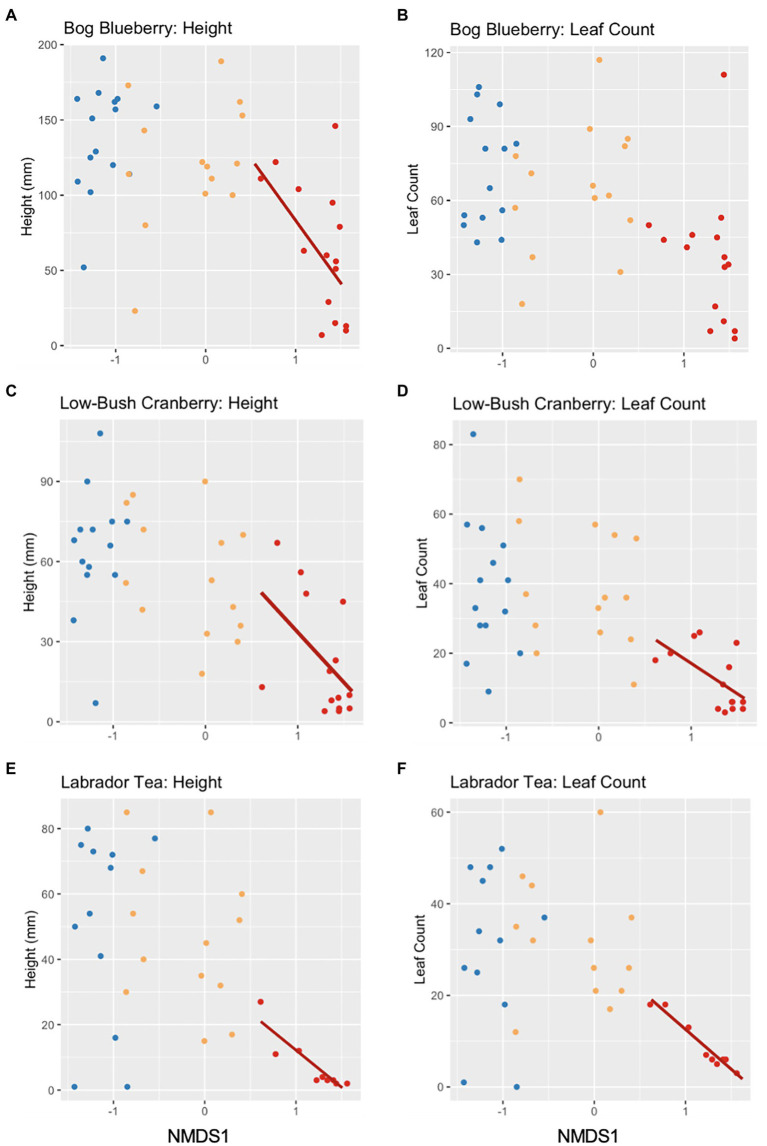
Scatterplots showing the effects of microbial community variation and plant productivity (16S rRNA amplicon NMDS1 and height/leaf count) of bog blueberry **(A,B)**, low-bush cranberry **(C,D)**, and Labrador tea **(E,F)**. Each point represents a soil community, colored by the level of FPES soil disturbance with blue = undisturbed (UD; *n* = 16), gold = semi disturbed (SD; *n* = 15), and red = most disturbed (MD; *n* = 16). Solid lines represent a significant linear relationship.

Following observing significant changes in microbial community diversity depending on the level of soil disturbance, we sought to determine whether variation within disturbance level community composition could predict the growth of plants from an earlier experiment described in Seitz et al. (2021). Within each disturbance level we regressed the productivity measures (height or leaf count) against the NMDS1 values for each corresponding microbial community used as an inoculant in the plant growth experiment (Seitz et al., 2021). We observed significant negative linear relationships between MD microbial community composition and plant growth within bog blueberry height, low-bush cranberry height and leaf count, and Labrador tea height and leaf count (Table 3; Figure 4). For bog blueberry, low-bush cranberry, and Labrador tea plants grown with MD microbes, plant growth continued to decrease as microbial communities became more extreme and more positive along the NMDS1 axis (NMDS1 > 0.5. Microbial community composition within SD and UD communities was not a significant predictor of height or leaf count in any plant species except for black spruce (height) from Seitz et al., 2021; Table 3).

**Table 3:** Results from linear models of plant growth measures predicted by 16S NMDS1.

Plant	FPES level	Growth measure	Estimate	SE	*t* Value	*p* Value	BH adjusted *p* value
BB	UD	Height	47.4	39.0	1.21	0.246	0.486
	SD	Height	27.0	23.0	1.17	0.264	0.486
	**MD**	**Height**	**−83.5**	**36.7**	**−2.27**	**0.041**	**0.244**
	UD	Leaf count	13.4	36.4	0.37	0.719	0.719
	SD	Leaf count	14.6	14.3	1.02	0.329	0.486
	MD	Leaf count	−21.9	25.5	−0.86	0.405	0.486
CB	UD	Height	23.8	37.9	0.63	0.542	0.542
	SD	Height	−17.6	12.1	−1.45	0.172	0.344
	**MD**	**Height**	**−38.5**	**18.6**	**−2.07**	**0.06**	**0.181**
	UD	Leaf count	−26.2	30.7	−0.85	0.411	0.493
	SD	Leaf count	−9.6	9.5	−1.02	0.33	0.493
	**MD**	**Leaf count**	**−17.3**	**7.0**	**−2.48**	**0.029**	**0.175**
LT	UD	Height	4.2	36.0	0.12	0.909	0.948
	SD	Height	−11.0	13.4	−0.82	0.429	0.858
	**MD**	**Height**	**−23.4**	**4.2**	**−5.57**	**0.001**	**0.003**
	UD	Leaf count	1.4	21.2	0.07	0.948	0.948
	SD	Leaf count	−3.5	8.0	−0.44	0.669	0.948
	**MD**	**Leaf count**	**−17.4**	**1.6**	**−11.14**	**<0.001**	**<0.001**
BS	**UD**	**Height**	**37.7**	**16.6**	**2.27**	**0.04**	**0.238**
	SD	Height	−7.3	8.3	−0.88	0.396	0.756
	MD	Height	7.3	11.7	0.62	0.543	0.756
	UD	Leaf count	70.8	47.3	1.50	0.156	0.469
	SD	Leaf count	−10.0	27.1	−0.37	0.721	0.756
	MD	Leaf count	18.7	59.2	0.32	0.756	0.756
FW	UD	Height	3.4	25.4	0.13	0.896	0.896
	SD	Height	−31.6	27.6	−1.14	0.277	0.613
	MD	Height	9.4	22.6	0.42	0.682	0.833
	UD	Leaf count	−6.2	5.8	−1.07	0.306	0.613
	SD	Leaf count	−1.7	4.2	−0.40	0.694	0.833
	MD	Leaf count	−6.2	3.4	−1.82	0.091	0.545

Bold denotes significant Benjamini-Hochberg (BH; Benjamini and Hochberg, 1995) adjusted *p* value < 0.25.

### Differences in Community Function Based on Soil Disturbance Level

**Figure 5:**
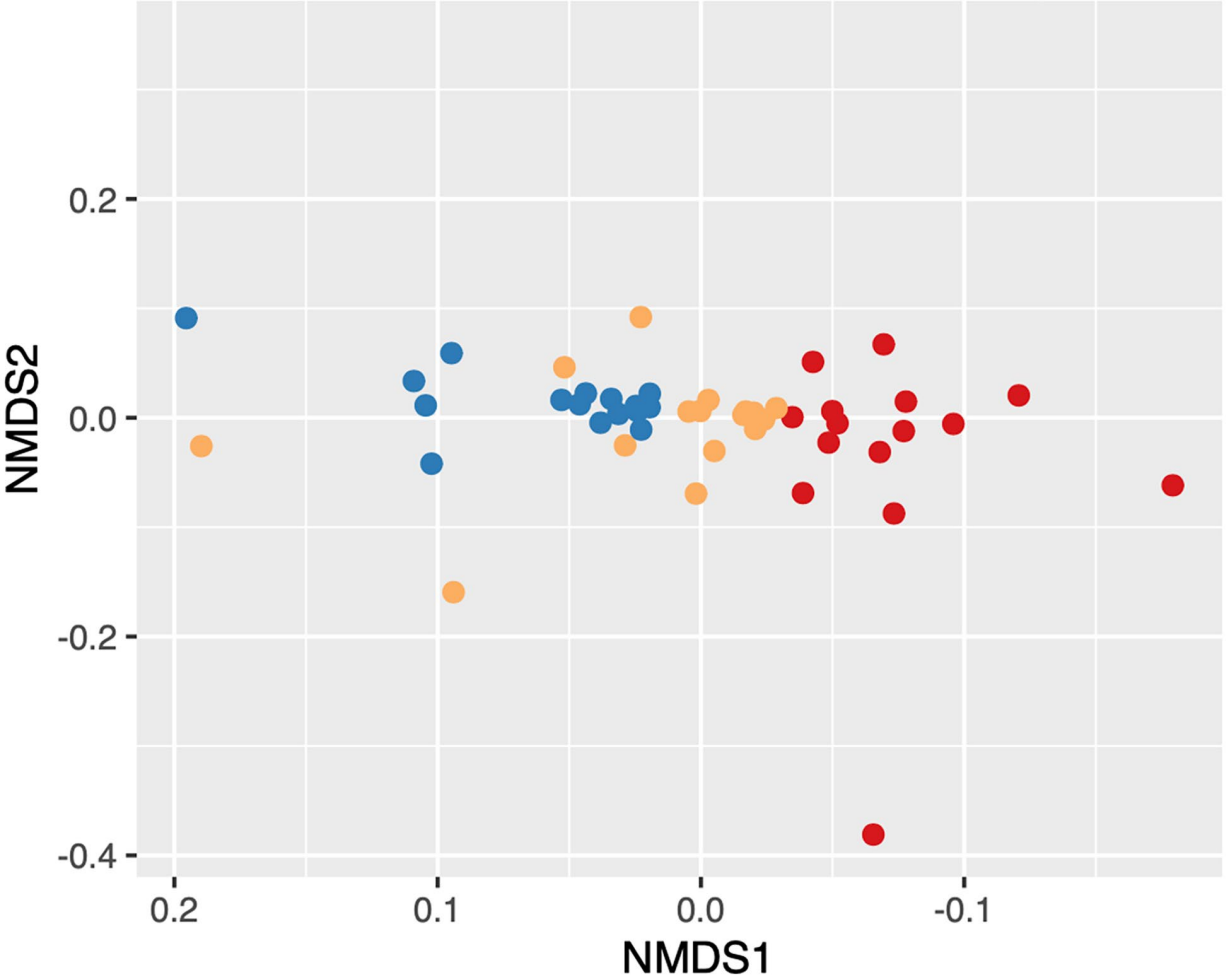
NMDS of Bray–Curtis dissimilarity distances of community function. Functions based on MEGAN-LR evolutionary genealogy of genes: Non-supervised Orthologous Groups (EGGNOG) Clusters of Orthologous Group (COG) classifications. Points colored by the level of FPES soil disturbance with blue = undisturbed (UD; *n* = 16), gold = semi disturbed (SD; *n* = 15), and red = most disturbed (MD; *n* = 16).

Based on the observed EggNOG annotations from our metagenomic data, we identified 2,891 clusters of orthologous genes (COGs) and 7,980 non-supervised orthologous groups across all samples. Using Bray-Curtis distances and a PERMANOVA analysis, we found that potential community function varied significantly based on the level of soil disturbance (Figure 5; stress = 0.14; PERMANOVA *F*_2,44_ = 0.17431, *p* = 0.001).

To identify specific functions that are characteristic of the three levels of soil disturbance, we performed an indicator analysis on the observed potential genomic functions of our microbial communities. After filtering the results for a high specificity (*A* ≥ 0.8) and sensitivity (*B* ≥ 0.5), we found 14 COG indicators, with 11 belonging to MD, two SD, and one UD (Supplementary Figure S1; Table 4). Within the indicator COGs, we identified a variety of clusters. The only COG identified from UD encodes for a chloramphenicol acetyltransferase an enzyme involved in the detoxification of the antibiotic chloramphenicol associated with resistance to chloramphenicol in bacteria. The COGs from SD were associated with basal transcription factors and nucleic acid binding. From MD, we identified a variety of COGs including those encoding for a for alpha-N-arabinofurandase (ABF), a cell-wall degrading enzyme, disulfide reductases, and a hydrogenase iron–sulfur subunit involved in methanogenesis and a nitrate reductase beta subunit necessary in denitrification.

**Table 4:** Highly sensitive and specific indicator functions from EggNOG annotations.

FPES	COG	Sensitivity (A)	Specificity (B)	Statistic	*p* Value	Function
UD	COG4845	0.8029	0.875	0.838	0.001	Chloramphenicol O-acetyltransferase
SD	COG5163	0.9804	0.5	0.7	0.001	Component of the NOP7 complex
SD	COG5033	0.852	0.5	0.653	0.001	Myeloid lymphoid
MD	COG3940	0.9239	0.625	0.76	0.001	Alpha-N-arabinofurandase, hydrolase activity
MD	COG5598	0.8921	0.75	0.818	0.001	Trimethylamine methyltransferase
MD	COG1423	0.88	0.5625	0.704	0.001	ATP dependent DNA ligase
MD	COG4305	0.8765	0.5625	0.702	0.001	Rare lipoprotein a
MD	COG1908	0.8745	0.6875	0.775	0.001	Methyl-viologen-reducing hydrogenase, delta subunit
MD	COG4559	0.8584	0.625	0.732	0.001	Part of the ABC transporter complex HmuTUV
MD	COG1140	0.8409	0.75	0.794	0.001	Nitrate reductase beta subunit
MD	COG3487	0.8401	0.625	0.725	0.002	Iron-regulated protein
MD	COG1148	0.8199	0.8125	0.816	0.001	CoB--CoM heterodisulfide reductase activity
MD	COG4452	0.806	0.5	0.635	0.007	Cell envelope integrity protein *CreD*
MD	COG2231	0.8051	0.5	0.634	0.009	Hhh-gpd family

## Discussion

This study provides a thorough microbial analysis including determining differences in microbial community composition at a fine taxonomic resolution, in alpha diversity, and in microbial function based on indicator species analysis results. Combined with the findings in our previous study (Seitz et al., 2021), our data give a more comprehensive picture of how disturbance affects microbial communities and mediates changes in plant communities.

Supporting our hypothesis that soil disturbance drives community shifts; we found that microbial community composition significantly differed with level of soil disturbance at the ASV level (Figures 1, 2) despite within site heterogeneity especially in UD and SD. This is consistent with our previous findings that community composition differed across these three disturbance levels (Seitz et al., 2021). We continued to look at these shifts in taxonomic composition through the lens of biomarkers previously identified in our metagenomic data as either over- or underrepresented in UD and MD communities (Seitz et al., 2021). We found that the relative abundances of these biomarkers were significantly positively correlated between our 16S and metagenomics datasets, further underlining that long read metagenomic sequences are a useful tool to assess differences in microbial community composition across different environmental conditions (Seitz et al., 2021).

We detected significant increases in alpha diversity with increasing in soil disturbance. This is a phenomenon that is observed within many ecosystems subject to many different types of disturbance, suggesting that disturbance promotes diverse communities due to the creation of new niches within the environment (Violle et al., 2010; Galand et al., 2016). Our findings of microbial community diversity increasing with disturbance are consistent with long-term (Feng et al., 2020) and short-term (Luláková et al., 2019) studies of the effects of warming soils and permafrost thaw on active layer bacterial communities. Feng et al. (2020) and Luláková et al. (2019) both saw alpha diversity measures significantly increase as topsoil temperatures increased. This trend of alpha diversity increasing along with soil disturbance has also been demonstrated in soils from a variety of land and forest types that have been subject to physical disturbances, such as logging, agricultural conversion, and fires (Chatterjee et al., 2008; de Carvalho et al., 2016; Shen et al., 2016; Zhou et al., 2018). The physical soil disturbance and resulting deep permafrost thaw that the MD level plot underwent has created a heterogenous landscape (Douglas et al., 2008), which may be contributing to this increased diversity by affecting the ecological niches and competition ability of bacteria for resources, similar to what can occur after forest fires (Shen et al., 2016).

We observed a lower community evenness that within our UD communities compared to our MD and SD communities. The relative abundance of Acidobacteria decreased drastically while relative abundance of Nitrosomanadaceae and Comamonadaceae increased with increasing soil disturbance. When ASVs were grouped at the family level, *Acidobacteriaceae* was the most abundant bacterial family within the UD communities, compared to being nearly absent in MD communities. Members of *Acidobacteriaceae* are typically found to be present at higher abundances in acidic soils that are carbon poor (Kielak et al., 2016; Osburn et al., 2019). In contrast, *Nitrosomonadaceae*, a copiotrophic bacterial family of ammonia oxidizers, was found to be the most abundant family within the MD soil communities, followed by *Comamonadaceae*. *Nitrosomonadaceae* are ammonia oxidizing bacteria (AOB) that are commonly found in carbon-rich environments such as wastewater and soils (Prosser et al., 2014). An increase of copiotrophic bacteria with disturbance is consistent with the body of literature (Masse et al., 2017; Mickan et al., 2019; Osburn et al., 2019). Masse et al. (2017) previously detected that oligotrophic bacteria, such as *Acidobacteriaceae*, were more abundant in natural boreal forest soils, compared to soils that were reclaimed or reconstructed after disturbance which displayed higher abundances of copiotrophic bacteria.

We found that the microbial community composition across soil disturbance is a significant predictor of plant productivity measures for bog blueberry (*Vaccinium uliginosum*), low-bush cranberry (*Vaccinium vitis-ideae*), and Labrador Tea (*Rhododenron groenlandicum*). Specifically, we can predict that plants inoculated with a microbial community indicative of the most extreme disturbance, will demonstrate decreased growth compared to plants inoculated with communities from less disturbed soils. This significant relationship between microbial community composition and plant productivity was observed within plants inoculated with MD microbes, whereas we saw no significant relationships between plant productivity of plants inoculated with microbial communities from SD or UD and microbial community composition. This suggests that there is a threshold effect occurring across the disturbance levels, and that soil microbial communities subject to disturbance resulting in permafrost thaw may not have the capacity to influence plant productivity of these boreal species until affected by an event of large enough magnitude. This microbial threshold we are observing could potentially be linked to a disturbance threshold of deep permafrost thaw. Over the past 20 years, researchers have observed accelerated permafrost thaw within the northern boreal Taiga plains likely due to a combination of hydro-climactic changes and increases in annual air temperatures (Jorgenson et al., 2006; Lara et al., 2016; Chasmer and Hopkinson, 2017). As permafrost thaw is accelerated and other disturbance events are increasing in the artic, we may start to observe the effects through changes in soil microbial communities and plant productivity.

Our previous study (Seitz et al., 2021) lacked any analysis of community function, and based on the disturbance-associated community membership shifts we observed, we predicted that observed functional community will shift as well. Using the metagenomic sequencing data, we identified functional indicators across all our FPES sites representative of each level of soil disturbance, with most indicators belonging to MD soils. The gene cluster with the highest specificity within MD is COG3940, which encodes for alpha-N-arabinofurandase (ABF). ABFs are mainly extracellular enzymes that degrade lignocelluloses and cell walls (De Ioannes, 2000; Numan and Bhosle, 2006), and have been shown to play a role in triggering plant immune responses. In 2016, Wu et al. found that a novel ABF plays a critical role in the pathogenicity of the fungal pathogen, *Magnaporthe oryzae*, that causes rice blast disease. The secreted ABF degrades cell walls, further leading to a decrease in productivity in infected plants (Wu et al., 2016). The presence of a gene cluster encoding for alpha-N-arabinofurandase in our MD communities provides a possible mechanism behind the decrease in plant productivity demonstrated in Seitz et al. (2021), yet further studies involving gene expression and enzyme activity would be required to identify if this is part of a mechanism at play.

The critical gene involved in ammonia oxidization (*amo*A; Prosser et al., 2014; Bárta et al., 2017) was present across all our sample communities, however, this gene cluster was not identified as a functional indicator of MD communities where *Nitrosomonadaceae* were very abundant. Curiously, we identified COG1140, a gene cluster encoding for a nitrate reductase beta subunit indicative of denitrification, to be a highly specific and significant indicator of MD soil communities. Nitrate losses within soils have long been known to follow disturbance events in forests such as fire, disease, or clear cutting (Vitousek et al., 1979; Neary et al., 1999; Hobara et al., 2001; Pardo et al., 2002). However, comparatively little is known about denitrification in the boreal system. This potential loss of available nitrate could be part of the mechanism at play in the reduced plant productivity that Seitz et al. (2021) observed within plants grown in MD community inoculated soils.

As climate change and anthropogenic-driven soil disturbance events including wildfires (Calef et al., 2008; Chapin et al., 2008; Partain et al., 2016) and permafrost thaw (Jorgenson et al., 2006; Lara et al., 2016; Chasmer and Hopkinson, 2017) are increasing across the north, we need to continue to study how disturbance influences the microbiomes of active layer soils that are both directly and indirectly involved in mediating plant structure within boreal forests. In this study, we show that active layer soil microbial communities are influenced by the initial disturbance event and permafrost thaw at FPES, however we cannot conclude whether specific environmental factors are associated with community shifts. We observed significant shifts in microbial community composition, in microbial diversity, as well as microbial community function. Our evidence for microbial community composition being a significant predictor of decreased plant productivity in response to the most severe soil disturbance associated with significant permafrost thaw suggests that this effect may occur once a threshold level of disturbance is reached. These consistent effects of disturbance on active layer microbial communities, combined with the knowledge of how they in turn can significantly affect plant productivity (Seitz et al., 2021), highlight the need for more research into how on-going and future disturbance events will influence the ecological dynamics in boreal forests.

## Data Availability Statement

The original contributions presented in the study are publicly available. This data can be found at: PRJEB42020.

## Author Contributions

US and DD contributed to the conception, design, and analysis of the study. TS performed the data collection, statistical analysis and wrote the first draft of the manuscript. All authors contributed to the article and approved the submitted version.

## Funding

Research reported in this publication was supported by an Institutional Development Award (IDeA) from the National Institute of General Medical Sciences of the National Institutes of Health under grant number P20GM103395 as well as under three linked awards numbers RL5GM118990, TL4GM118992, and 1UL1GM118991.

## Conflict of Interest

The authors declare that the research was conducted in the absence of any commercial or financial relationships that could be construed as a potential conflict of interest.

## Publisher’s Note

All claims expressed in this article are solely those of the authors and do not necessarily represent those of their affiliated organizations, or those of the publisher, the editors and the reviewers. Any product that may be evaluated in this article, or claim that may be made by its manufacturer, is not guaranteed or endorsed by the publisher.

## Footnotes

^1^ https://earthmicrobiome.org/protocols-and-standards/16s/

^2^ https://benjjneb.github.io/dada2/index.html
