# Supplementary material for: Unearthing Shifts in Microbial Communities Across a Soil Disturbance Gradient
Source: Front Microbiol. 2022 May 24;13:781051. doi: 10.3389/fmicb.2022.781051 (PMC9171198; doi:10.3389/fmicb.2022.781051)
Supplement: Supplementary file 1 [file Data_Sheet_1.docx]

Supplementary Material

**
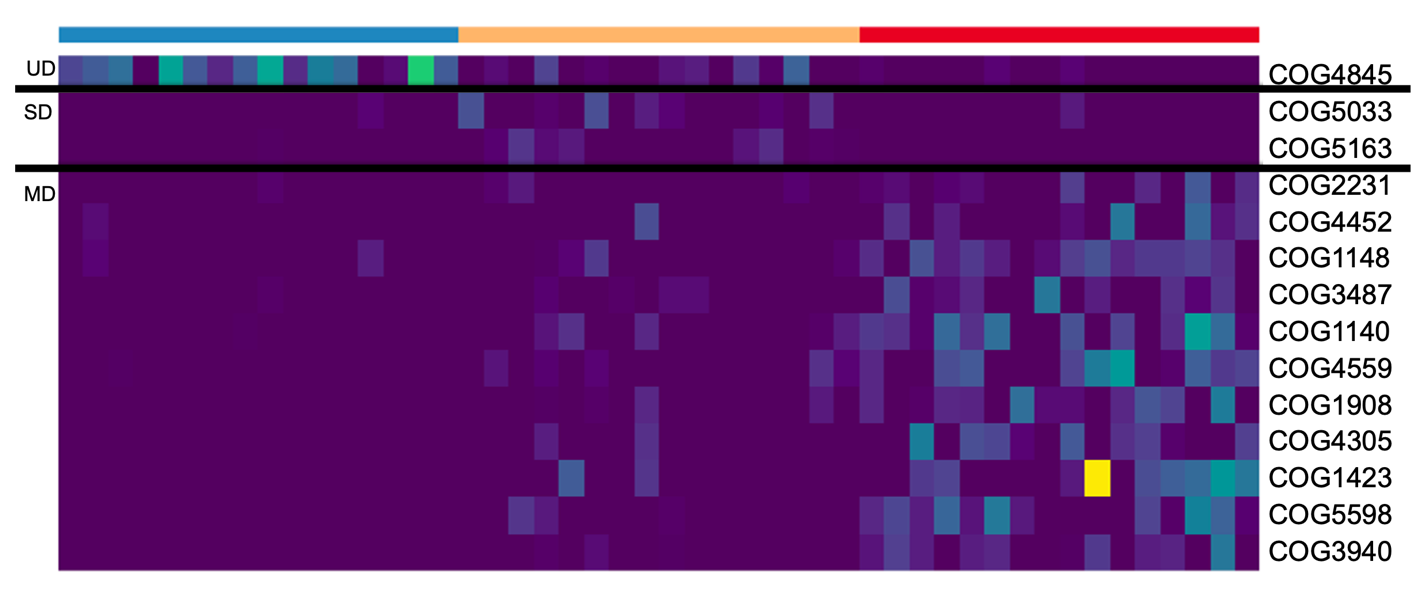
**

**Figure S1.** Heatmap of highly sensitive and specific indicator functional EggNOG annotations

from metagenomic data. Each row corresponds to a COG, and each column corresponds to an individual soil core community. The top row signifies the level of soil disturbance of each core with blue = UD (n = 16), gold = SD (n = 15), and red = MD (n = 16). The color of each box corresponds to the normalized read count of the COG within each core.
